# Nanotopography Alters Nuclear Protein Expression, Proliferation and Differentiation of Human Mesenchymal Stem/Stromal Cells

**DOI:** 10.1371/journal.pone.0114698

**Published:** 2014-12-18

**Authors:** Karina Kulangara, Jennifer Yang, Malathi Chellappan, Yong Yang, Kam W. Leong

**Affiliations:** Department of Biomedical Engineering, Duke University, Durham, North Carolina, United States of America; The University of Adelaide, Australia

## Abstract

Mesenchymal stem/stromal cells respond to physical cues present in their microenvironment such as substrate elasticity, geometry, or topography with respect to morphology, proliferation, and differentiation. Although studies have demonstrated the role of focal adhesions in topography-mediated changes of gene expression, information linking substrate topography to the nucleus remains scarce. Here we show by two-dimensional gel electrophoresis and western blotting that A-type lamins and retinoblastoma protein are downregulated in mesenchymal stem/stromal cells cultured on 350 nm gratings compared to planar substrates; these changes lead to a decrease in proliferation and changes in differentiation potential.

## Introduction

Mesenchymal stem/stromal cells (MSCs) are an attractive cell source for regenerative medicine applications. MSCs can be readily expanded *in vitro* and differentiated into the adipogenic, osteogenic, chondrogenic or myogenic lineage [1]–[3]. They can also modulate the immune response [4], [5]. MSC therapy often involves the use of a scaffold, either for expansion and differentiation *in vitro* or direct application *in vivo*. Scaffold properties such as topography and elasticity can influence the gene expression and differentiation of MSC [6]–[9]. Several recent studies have contributed to our understanding on how MSCs sense substrate topography at the molecular level [6], [10], however, how signal from the surrounding microenvironment is relayed to the nucleus and translated into gene expression changes is largely unknown.

Lamins form a meshwork at the inner nuclear membrane known as nuclear lamina [11]. The A-type lamins are encoded by the LMNA gene, which gives rise to the lamin A and lamin C proteins by alternative splicing. A large number of proteins bind to the nuclear lamina. These range from signaling proteins to chromatin and transcription factors [12]. Mutations of the LMNA gene give rise to at least nine clinically distinct diseases affecting primarily tissues of mesenchymal origin [13]–[20]. Lamins are important for the structural integrity of the nucleus and nuclear architecture as well as gene transcription [11]. pRB is one example of a transcription factor that binds to lamin A [21]. pRB regulates terminal differentiation and its interaction with A-type lamins is required for its stability, as pRB is rapidly degraded in *Lmna*-null mouse cells [22].

An interesting recent study describes that the level of A-type lamins correlates with tissue elasticity [23]. The authors showed that in cultured cells the expression of A type lamin is mechanosensitive and its turnover is inversely proportional to matrix elasticity. Moreover lamin-A levels directly or indirectly regulate proteins involved in tissue-specific gene expression and link tissue mechanics to changes in differentiation, development, injury and diseases.

We examine here how molecules in the nucleus are altered in response to substrate topography resulting in changes in gene expression. An understanding of how cell-topography interactions affects proliferation and differentiation may help design medical devices and biomaterials with specific functionality. Here we show by two-dimensional gel electrophoresis and western blotting that A-type lamins and retinoblastoma protein are downregulated in mesenchymal stem/stromal cells cultured on 350 nm topography gratings compared to planar substrates; these changes lead to a decrease in proliferation and alterations in differentiation potential.

## Materials and Methods

### Fabrication of PDMS substrates with 350 nm gratings or planar controls

Nanogratings of 350 nm linewidth, 700 nm pitch, and 280 nm depth were written on a poly(methylmethacrylate) (PMMA) thin film that was spin-coated onto a Si substrate using electron beam lithography (EBL) as previously described [24], [25]. Substrates with no features or 350 nm topography for cell culture were replicated in PDMS because of the ease of fabrication. To generate a master for larger nanopatterned surfaces multiple inverse PDMS molds were stitched together and used to hot-emboss onto a polystyrene (PS) thin film that was spin-coated on a Si wafer.

A mixture of PDMS resin and curing agent (SYLGARD 184 kit, Dow Corning, MI, USA) in a 10:1.05 w/w ratio was poured onto the EBL mold and degassed in a vacuum chamber for 60 min. After curing for 2 h at 65°C, and once the PDMS reached RT the inverse PDMS mold was peeled from the EBL mold. The PDMS substrate with nanogratings or planar PDMS was used for cell culture. PDMS substrates were sterilized by ethanol, followed by UV exposure for 30 min. For cell culture the PDMS substrates were coated with collagen I (BD Biosciences, Bedford, MA) at 15 µg/cm^2^ to enable hMSC attachment [26].

### Human mesenchymal stem/stromal cell culture

hMSCs (Tulane Center for Gene Therapy, New Orleans, LA) were cultured and expanded in Complete Culture Medium (CCM) with 10% fetal bovine serum according to the Tulane Center for Gene Therapy/Prockop lab protocol. hMSCs used in the experiments were at passage 3–6. The hMSCs were seeded on the 350 nm patterned or planar PDMS at 6×10^3^ cell/cm^2^. Planar PDMS was used as a control. The cell culture media was changed every 3 to 4 days.

### Immunofluorescent staining

Using the Click-iT EdU (5-ethynyl-2′-deoxyuridine) cell proliferation assay kit (Invitrogen, Eugene, OR), 2× EdU was added to the medium in a 1∶1 v/v ratio one day after seeding to allow the cells to recover overnight. Cells were cultured in the EdU-containing media for 2 or 6 days. For the 6 day conditions, 2× EdU was added whenever the medium was changed. Samples were fixed by incubating in 4% paraformaldehyde for 15 minutes and permeabilized with 0.5% Triton X-100 for 20 minutes. EdU detection was performed per Click-iT EdU Imaging kits protocol using Alexa Fluor 488 azide to label the EdU via a copper-catalyzed triazole reaction. 4′,6-diamidino-2-phenylindole (DAPI, Molecular Probes) was used as a counter stain for the nucleus. Samples were inspected by confocal microscopy.

### Protein extraction

hMSCs were detached from planar PDMS (control) or 350 nm gratings (sample) using a cell scraper in ice cold PBS after 7 days of culture and an initial wash in ice cold PBS. Cells were pelleted with a 5 minutes spin at 500 g at 4°C and lysed in focusing buffer (8 M urea, 4% CHAPS, 30 mM Tris-HCl, pH 8.5), and total protein concentration was determined with the 2D Quant kit (GE-Healthcare).

### Cy-dye labeling

The internal standard methodology was used to determine protein differential expression [27]–[29]: 120 µg of total protein was taken from control or sample and then labeled with 8 pmol/µg of Cy3 for control and Cy5 for sample, using a described protocol of the GE-Healthcare Kit (25800983). Additionally, 60 µg of control and sample were pooled and labeled with 8 pmol/µg of Cy2 to create the internal standard (IS) sample. The proteins were labeled for 30 min on ice in the dark. The labeling reaction was stopped with 1 µL of 10 mM lysine for 10 min on ice in the dark. Pooling samples minimizes sample-to-sample variations and allows for pair-wise comparisons in a single gel with different labels, minimizing gel-to-gel variations. The resulting volume was diluted with an equal volume of 2× sample buffer (8 M urea, 4% CHAPS, 20 mg / mL DTT, 2% v / v IPG (Immobilized pH Gradients) Buffer 3–10 (GE-Healthcare)) and placed on ice for 15 min. Samples were then supplemented with rehydration buffer (8 M urea, 4% CHAPS, 2 mg / mL DTT, 1% v / v IPG Buffer 3–10) to give a final volume of 250 µL.

### 2D gel electrophoresis and imaging

Labeled samples (250 µL) were applied to IPG strips (13 cm, pI ranges 3–10, GE-Healthcare) on the rehydration tray (GE-Healthcare) and focused using an Ettan IPGphor II (GE-Healthcare) as follows: active rehydration at 30 V for 14 h, followed by isoelectric focusing for a total of 28 kV/h (step to 500 V for 1 h, step to 1000 V for 1 h, step to 8000 V to a total of 28 kV/h). After isoelectric focusing, disulfide bonds were reduced by placing the strips for 10 min in 20 mL equilibration buffer (6 M urea, 50 mM Tris, pH 8.8, 30% glycerol, 2% SDS) containing 5 mg / mL DTT. The strips were then incubated for 10 min in fresh equilibration buffer with 45 mg / mL iodoacetamide. For the second dimension, the IPG strips were placed on 12% homogeneous polyacrylamide gels (4% stacking). Gels were cast using low-fluorescence glass plates (13 cm plates, GE-Healthcare) previously treated with bindsilane (GE-Healthcare). Each SDS-PAGE was run at 9 mA for 16 h in a HOEFER SE-600 system. Individual images of Cy2-, Cy3- and Cy5-labeled proteins of each gel were obtained using a Typhoon 9410 scanner (GE-Healthcare) with excitation / emission wavelengths of 480 / 530 nm for Cy2, 520 / 590 nm for Cy3 and 620 / 680 nm for Cy5. After imaging the gels were stained with colloidal Coomassie (Bio-Rad, Hercules, CA, USA).

### DIGE analysis

2D-DIGE gels were analysed with DeCyder 2D 6.5 software (GE-Healthcare) to identify proteins displaying differential expression levels. Comparisons of abundance changes were examined across three replicate experiments, and for pair-wise comparisons of individual Cy3–control and Cy5–time points. Two standard deviations from the mean volume ratios (95th percentile confidence) were used as threshold to determine levels of significance for a given set of samples. Statistical analysis and gel-to-gel comparison was performed with the Biological Variation Analysis (BVA) module (GE-Healthcare).

### Protein Identification

Protein spots that showed significant changes in levels were processed for protein identification by tandem mass spectrometry at the UNC-Duke Michael Hooker Proteomics Center of the University of North Carolina, Chapel Hill. Briefly, gel plugs were submitted to in-gel digestion with modified trypsin, and MALDI-MS / MS (matrix assisted laser desorption ionization-mass spectrometry) data were acquired using a 4700 Proteomics Analyser MALDI-TOF / TOF (matrix-assisted laser desorption ionization-time of flight; Applied Biosystems, Inc. (ABI), Framingham, MA, USA). MS and MS / MS peak spectra were acquired and the 15 most intense peaks with a signal-to-noise ratio greater than 20 were selected automatically for MS / MS analysis. The peptide mass fingerprinting and sequence tag data from the TOF / TOF were evaluated with GPS Explorer scores (ABI). The MS and MS / MS spectra were used by the Mascot search engine to identify proteins from non-redundant databases (NCBI, MSDB) [30]. The MALDI data and protein annotations were also manually verified against the GenBank translated protein database.

### RNA extraction and qPCR

Total RNA was isolated from hMSCs at 3 and 7 days after being grown on 350 nm patterned PDMS or planar PDMS as a control. RNA was isolated with a RNeasy Mini Kit according to manufacturer's protocol (Qiagen, Valencia, CA) and RNase-free DNase treatment performed to remove any traces of DNA. Total RNA samples were assessed with a Nanodrop 8000 spectrophotometer (Thermo Scientific/Nanodrop, Wilmington, DE). Comparative CT realtime reverse transcription-PCR was performed in 20 µL reactions using the QuantiTect SYBR Green RT-PCR Kit (QIAGEN, Valencia, CA) with 10 ng of starting RNA isolated with RNeasy and QIAshredder kits (QIAGEN). PCR proceeded for 40 cycles in an ABI 7900HT Real-Time PCR System (Applied Biosystems, Carlsbad, CA). Three independent experiments were performed in triplicates. Target gene expression levels were normalized to endogenous GAPDH references, and presented as a fold-change relative to expression levels from cells cultured on planar control substrates. The primers used in this study are listed in S1 Information.

### Protein expression profiles

For Western blot, hMSCs were cultured for 7 days on 350 nm patterned or planar PDMS and lysed in ice cold RIPA buffer (Teknova, Hollister, CA). Protein concentration was determined using the BCA kit (PIERCE, Rockford, IL) and 2× sample buffer (Sigma, St. Louis, MO) was added. Proteins were then separated in 4–20% Tris-HCL polyacrylamide gel in Tris/Glycine/SDS Buffer (BioRad, Hercules, CA). Proteins were transferred onto 0.2 µm nitrocellulose Ready Gel Blotting Sandwiches (BioRad) and Ponceau S Red solution (Sigma-Aldrich, St. Louis, MO) was used to confirm equal transfer of all samples. Retinoblastoma (Calbiochem, San Diego, CA) and Lamin C (Abcam, Cambridge, MA) was used as the primary antibody with GADPH (Abcam, Cambridge, MA) as the control primary. Secondary antibodies were goat anti-mouse IgG conjugated to horseradish peroxidase (Novagen, Madison, WI). Chemiluminescence was performed by Amersham ECL Plus Western blotting detection system (GE Healthcare, Chalfont St. Giles, UK) and imaged with the FluorChem Imaging system (Alpha Innotech, San Leandro, CA).

### Data analysis

All data are presented as mean ± SD when statistical analysis could be performed. Student's t-test was used to evaluate the statistical significance. Significance level was set at *p*<0.01.

## Results

**Figure 1:**
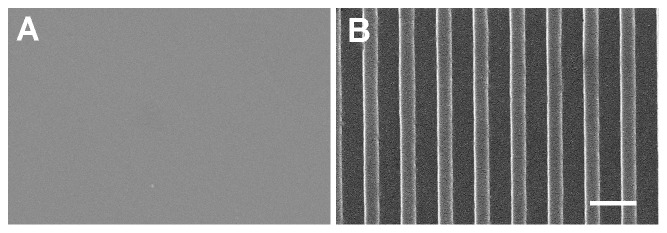
Scanning electron microscopy images of PDMS substrates used in this study. (**A**) Planar PDMS (**B**) PDMS substrate with 350 nm grating topography. Scale bar 1 µm.

**Figure 2:**
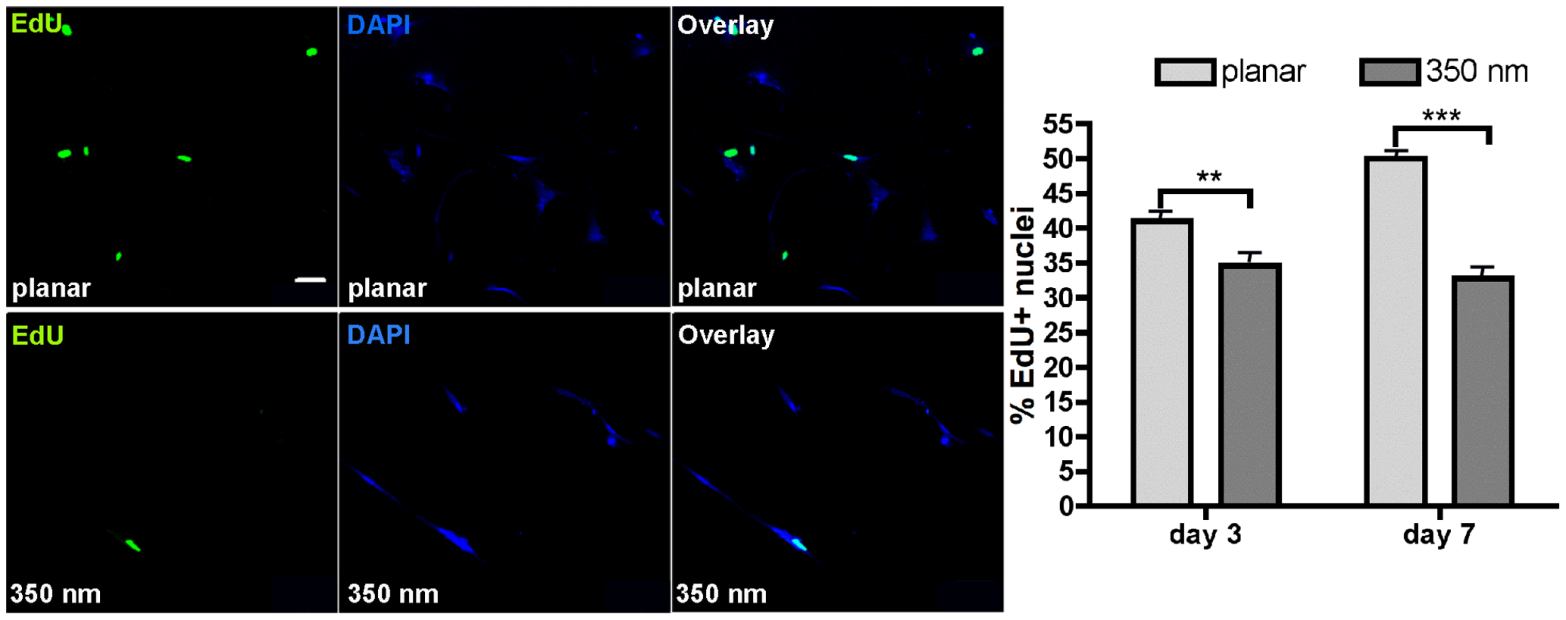
Proliferation of hMSCs on the planar and 350 nm patterned PDMS substrates. (**A**) Typical confocal micrographs of EdU^+^ nuclei, DAPI staining for total number of nuclei and the overlay images of hMSCs grown for 7 days on planar or 350 nm patterned PDMS. (**B**) Percent EdU stained nuclei compared to total nuclei, n>400 for each condition. ** p<0.048 and *** p<0.001.

We have previously reported a decrease in proliferation of hMSCs on 350 nm patterned PDMS compared to the planar PDMS over a short 4 hours of incubation with 5-bromo-2-deoxyuridine [6]. In order to understand in more details how cellular function is affected and to identify underlying mechanisms, we wanted to confirm the decrease in proliferation over a longer time course. We cultured hMSCs on planar control (Fig. 1A) or 350 nm grating substrates (Fig. 1B) for 3 or 7 days. The Click-iT EdU cell proliferation assay was used to assess proliferation because it is a less intrusive assay than BrdU (EdU does not require DNA denaturation as BrdU does) and allows for longer incubation periods. After 2 or 6 days of incubation with EdU, the proliferation of hMSCs was assessed by fluorescence microscopy and the presence of the green signal corresponding to incorporated EdU in the nucleus. Our results confirmed that on 350 nm patterned PDMS the proliferation was decreased compared to the proliferation on the planar PDMS (Fig. 2). In fact, the longer the cells were allowed to grow on the nanopatterned PDMS, the more pronounced was the difference in proliferation rates between the nanopatterned and planar PDMS conditions. At day 3, the decrease observed, 41.0±3.3% of EdU incorporation on the planar PDMS compared to 34.6±3.0% on the nanopatterned PDMS, was already significant (p<0.048). The difference in proliferation was even more striking when hMSCs were grown on the nanopatterned PDMS for 7 days, 32.8±3.1% of EdU incorporation, compared to 50.0±2.7% on the planar PDMS (p<0.001).

**Figure 3:**
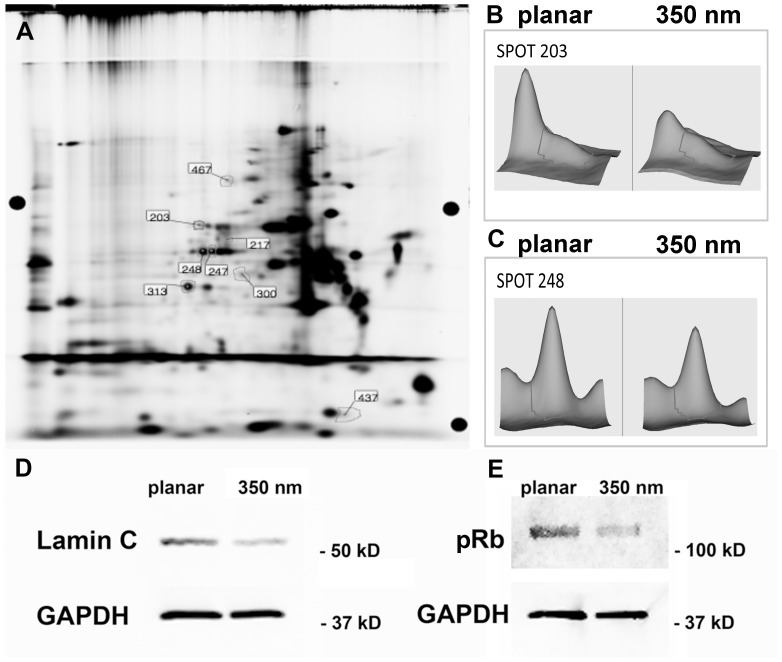
Analysis of the proteome in hMSC cultured on planar or 350 nm patterned PDMS substrates. (**A**) 2D-DiGE overlay image; 8 differentially expressed proteins were identified by 2D-DiGE. (**B**) Protein spot 203 was identified as Lamin A/C and (**C**) spot 248 as Lamin C by MALDI-MS/MS. (**D, E**) Western blot analysis confirmed the decrease in Lamin C and pRb expression in hMSC cultured on 350 nm grating topography.

We then screened for protein expression differences using two-dimensional differential in-gel expression (2D-DiGE). Significant changes in the proteome were detected in hMSCs cultured on 350 nm grating substrates compared to hMSC cultures on planar controls. On 350 nm gratings, 8 protein spots were differentially expressed (Fig. 3A). Of these, 7 protein spots were significantly down-regulated (range 16–44% below planar control levels) and one was significantly up-regulated as a function of nanogratings (range 54% above planar control levels). After mass spectroscopy (MS) analysis, 5 (of 8) spots were reliably identified with high confidence (>95%) scores. The inability to identify a spot was related to low amounts of protein in that particular spot, which in turn led to low confidence (<95%) protein identification by MS. Protein spot 248 was identified as Lamin C and spot 203 as Lamin A/C by MALDI-MS/MS (Fig. 3B, C). The A-type lamins make up the meshwork lining the nuclear envelope.

Although the 2D-DiGE approach we used was highly quantitative and is known to yield only a low percentage of false-positive detections, we chose to confirm the differential regulation of selected proteins of interest using Western blotting. The Western blot of hMSCs whole cell lysate with anti-Lamin C antibody showed a clear band in hMSC on planar substrate and a weaker band corresponding to a decrease in Lamin C expression normalized to GAPDH in hMSC cultured on 350 nm topography substrates (Fig. 3D).

As retinoblastoma protein plays a critical role in cell cycle control, differentiation and proliferation, and interaction with the A-type lamins we subsequently analyzed by Western blot the expression of Retinoblastoma (Rb) protein. As with Lamin C we observed a significant downregulation of Rb on 350 nm gratings compared to the planar control (Fig. 3E). These data indicate that 350 nm gratings influence the expression of Lamin C and Rb in the nucleus.

**Figure 4:**
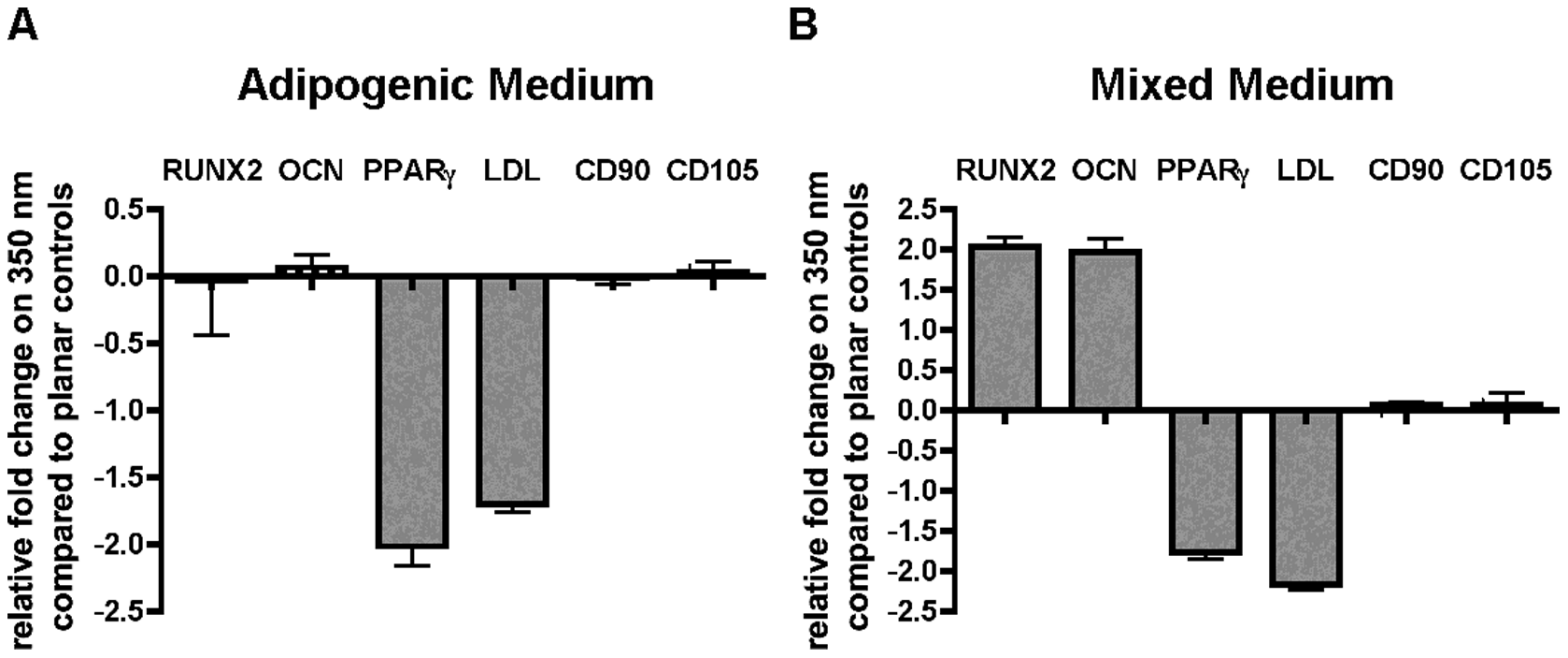
Nanotopography acts in synergy with biochemical cues to alter differentiation potential of hMSC. (**A**) Adipose differentiation is decreased on 350 nm topography when induced with adipose induction medium. Adipose specific genes *PPARγ*, and *LDL* are significantly downregulation in cultures on 350 nm topography. (**B**) In presence of mixed media conditions with biochemical cues promoting adipogenic and osteogenic differentiation, 350 nm topography increased osteogenic differentiation as shown by the upregulation of osteogenic genes *Runx2* and *Osteocalcin* and hindered adipogenic differentiation as shown by the decrease of adipogenic genes *PPARγ*, and *LDL* compared to hMSC cultured on planar control substrates.

The decrease in proliferation and the changes in Lamin C and Rb expression point to the possibility that 350 nm topography influences the differentiation potential of hMSCs. To test this hypothesis we differentiated hMSCs on planar and 350 nm topography first in presence of adipogenic media and assessed the gene expression by real-time quantitative PCR. The expression level of osteogenic markers *Runx2* and *Osteocalcin* (*OCN)* and markers of non-differentiated hMSCs *CD90* and *CD 105* were not significantly altered in hMSC cultured on 350 nm topography compared to control cultures on planar substrates. The adipogenic markers *PPARγ* and *LDL*, however, were significantly downregulated on 350 nm topography compared to planar controls. (Fig. 4A). We then cultured the hMSCs in mixed media containing adipogenic and osteogenic cues as described previously [31], [32] and assessed osteogenic and adipogenic gene expression. We observed again a decrease in the adipogenic markers *PPARγ* and *LDL* in hMSC cultured on 350 nm topography compared to control cultures on planar substrates. In contrast, the expression of osteogenic markers *Osteocalcin* and *Runx2* increased significantly in hMSC cultured on 350 nm topography compared to those on planar controls (Fig. 4B).

## Discussion

Here we report for the first time a link between the decrease in proliferation, the change in differentiation potential and changes in protein expression of A-type lamins and pRB in response to underlying nanotopography. Our results are interesting in several aspects: Firstly, we report a decrease in A-type lamin expression in response to substrate nanogratings compared to planar control substrates similar to the scaling of A-type lamins in response to soft substrates recently reported by Swift et al. [23]. This could suggest that hMSCs respond to soft substrates and nanogratings by similar molecular mechanisms. We have identified in the past another change on the level of zyxin protein expressed in focal adhesions of hMSCs cultured on 350 nm gratings compared to planar controls [10]. Interestingly the change in magnitude and trend of the turnover of zyxin in these hMSCS mirrors that of bovine adrenal capillary endothelial cells cultured on soft polyacrylamide gels of 50 KPa compared to those on 3.5 MPa [33]. The current results are another indication that changes in hMSC function in response to soft substrates or nanogratings may utilize the same molecular pathways. However, our results contrast with the ones described by Swift et al. by the fact that they have shown a shift towards adipogenic differentiation, whereas we have shown the opposite trend in response to underlying nanotopography. This discrepancy could be explained by the difference in media formulation used in the studies. We have used here a mixed medium formulated for adipogenic and osteogenic differentiation, whereas they use a proliferation medium in their study. Previous reports using mixed media formulation have also reported a decrease in adipogenic differentiation when hMSC cultured on rectangular surfaces adopt a more elongated phenotype [32], we have also previously reported such a morphology in response to the nanogratings used in this study [34]. Taken together these results confirm that physical cues in the form of substrate nanotopography, geometric restriction or soft substrates act in synergy with biochemical cues in the media to influence hMSC's differentiation potential [31]. Secondly, we report that pRb expression level is decreased by nanotopography. pRB controls cell lineage specification and aspects of differentiation by diverse mechanisms such as interacting with tissue-specific transcription factors, enhancing RNA interference, and modifying chromatin structure [35]. Our findings suggest that the expression of lamins and pRb may be linked and at the origin of the change in hMSC's differentiation potential. pRb activates among other functions target genes and transcription factors. There is a tremendous interest in activating and suppressing transcription factors in the reprogramming community and being able to do so with physical cues such as nanotopography is promising. Cell-substrate interactions are central to many biological processes. This work adds to the understanding of how cell-topography interactions may influence the phenotypes of hMSC, one of the most important cell types in regenerative medicine. The ability to manipulate cell fate and function via implant design is attractive as this can be exploited for either enhancing tissue regeneration or minimizing tissue reaction. The mechanistic understanding gained in this study is another step toward a more rational design of implants or scaffolds for various biomedical applications. In conclusion we show here how substrate nanotopography alters the expression of nuclear proteins lamin C and pRB, which then leads to changes in proliferation and differentiation of hMSC. This is an interesting new finding linking extracellular physical cues to changes in the nucleus.

## Supporting Information

S1 Information **The list shows the primers used in this study.**
(DOCX)

S2 Information **The following proteins were found to be differentially expressed in hMSCs in response of culture on 350 nm gratings compared to planar control.**
(DOCX)
