# Supplementary material for: Nanotopography Alters Nuclear Protein Expression, Proliferation and Differentiation of Human Mesenchymal Stem/Stromal Cells
Source: PLoS One. 2014 Dec 18;9(12):e114698. doi: 10.1371/journal.pone.0114698 (PMC4270691; doi:10.1371/journal.pone.0114698)
Supplement: S1 Information — The list shows the primers used in this study. (DOCX) [file pone.0114698.s001.docx]

**Information S1:**

Runx2 forward: TGGTTACTGTCATGGCGGGTA

Runx2 reverse: TCTCAGATCGTTGAACCTTGCTA

OCN forward: CGCAGCCACCGAGACACCAT

OCN reverse: AGGGCAAGGGGAAGAGGAAAGAA

LDL forward: CGACAGATGCGAAAGAAACGA

LDL reverse: CCCGGATTTGCAGGTGACA

CD 90 forward: GAGCCTTCGTCTGGACTGCCGC

CD 90 reverse: GCTCAGGCACCCCCACAGTGC

CD 105 forward: CTGCACGGTAGCCCTGCGTCC

CD 105 reverse: CTGGGGGAACGCGTGTGCGA
