# Supplementary material for: Nanotopography Alters Nuclear Protein Expression, Proliferation and Differentiation of Human Mesenchymal Stem/Stromal Cells
Source: PLoS One. 2014 Dec 18;9(12):e114698. doi: 10.1371/journal.pone.0114698 (PMC4270691; doi:10.1371/journal.pone.0114698)
Supplement: S2 Information — The following proteins were found to be differentially expressed in hMSCs in response of culture on 350 nm gratings compared to planar control. (DOCX) [file pone.0114698.s002.docx]

**Information S2:**

| AF080572 |
| --- |
| Lamin A/C transcript variant 1.- Homo sapiens |
| lamin C - human |
| T-complex protein 1 subunit beta (TCP-1-beta) |
| Alpha-enolase (2-phospho-D-glycerate) |
